# Comparison of axial-rotational postoperative periprosthetic fracture of the femur in composite osteoporotic femur versus human cadaveric specimens: A validation study

**DOI:** 10.1177/09544119221092842

**Published:** 2022-05-21

**Authors:** Jonathan N Lamb, Oliver Coltart, Isaiah Adekanmbi, Hemant G Pandit, Todd Stewart

**Affiliations:** 1 Leeds Institute of Rheumatic and Musculoskeletal Medicine (LIRMM), School of medicine, University of Leeds, Leeds, UK; 2 School of Mechanical Engineering, University of Leeds, Leeds, UK; 3 DePuy International, Johnson and Johnson, Leeds, UK

**Keywords:** Hip protheses, hip biomechanics, fracture, periprosthetic fracture, femur, sawbone, cadaver

## Abstract

Postoperative periprosthetic femoral fracture following hip replacement has been the subject of many varied experimental approaches. Cadaveric samples offer realistic fit and fracture patterns but are subject to large between-sample variation. Composite femurs have not yet been validated for this purpose. We compared the results of composite femurs to cadaveric femurs using an established methodology. In vitro postoperative periprosthetic fracture results using axial-rotational loading were compared between 12 composite femurs and nine fresh frozen femurs, which were implanted with an otherwise identical collarless (6 composite vs 4 cadaveric) or collared (6 composite vs 5 cadaveric) cementless femoral stem using identical methodology. Fracture torque and rotational displacement were measured and torsional stiffness and rotational work prior to fracture were estimated. Fractures patterns were graded according to the Unified Classification System. Fracture torque, displacement, torsional stiffness and fracture patterns for cadaveric and composite femurs were similar between groups. There was a trend for a greater rotational displacement in the cadaveric groups, which lead to a decrease in torsional stiffness and a significantly greater rotational work prior to fracture for all cadaveric specimens (collarless stems: 10.51 [9.71 to 12.57] vs 5.21 [4.25 to 6.04], *p *= 0.01 and for collared stems: 15.38 [14.01 to 17.05] vs 5.76 [4.92 to 6.64], *p* = 0.01). Given comparable fracture torque and the similarity in fracture patterns for fracture trials using composite samples versus cadaveric femurs, the use of composite femur models may be a reasonable choice for postoperative periprosthetic femoral fracture studies within certain limitations.

## Introduction

Postoperative periprosthetic fracture of the femur following total hip replacement (PFF) with a cementless femoral stem occurs in up to 5% of cases^1,2^ and has an associated 1 year mortality of 11 to 13%.^3,4^

The risk of PFF is strongly associated with a range of cementless stem implant characteristics^5–9^ and the study of contribution of stem design features has gained in popularity over the past decade. Studies use a range of methodology with the choice of specimen largely between composite femur^10–13^ and cadaveric specimens.^5,6,14,15^ The majority of studies, regardless of specimen choice use fracture loads (axial load or torque) at the moment of fracture as the primary outcome.^16^

Cadaveric samples can offer realistic implant fit, loading and fracture patterns but these benefits are offset by the large between-sample variation which occurs when testing groups of cadaveric specimens. Variability can be overcome using pairwise comparison of results in bilateral femur pairs,^5,6^ but this limits the experiment to the testing of one variable per pair of femurs. Composite femurs are a valid and highly uniform specimen choice when comparing whole bone composite femurs to whole bone cadaveric specimens^16,17^and do not require ethical approval or specialised handling and storage techniques to use effectively. However, validation has largely focused on the mechanical properties of the complete femur and validation to date relies on a single study comparing composite femur models to a single cadaveric femur trial.^13^

In this study we aimed to compare in-vitro results of PFF simulated methods using composite femur specimens to results using cadaveric specimens using identical loading protocols.

## Methods

To assess the validity of a composite femur model, results from tests using fresh frozen femur specimens were compared to results from tests using an osteoporotic femur composite model. Specimens from the fresh frozen cadaver trials have been published previously as part of a separate study.^6^

### Specimen preparation

#### Cadaveric specimens

This study was performed in accordance with local ethical guidelines and regulations of Hamburg University School of Medicine and the University of Leeds. Fresh frozen human female femora were dissected within 48-hours post-mortem, frozen at −20°C (2 freeze-thaw cycles per specimen), defrosted overnight before biomechanical testing and kept wet with saline solution and impermeable covers.

Preparation was been previously described^6^ and specimens were stripped of soft tissue and underwent computer tomography scanning (CT, Brilliance 16 CT; Philips Healthcare, Hamburg, Germany) to screen for pre-existing bony pathology which would bias results and to estimate bone mineral density.

#### Composite femur preparation

Composite femurs (Osteoporotic femur, 10 PCF Solid Foam core with 16 mm Canal, Medium, SawBones, WA) contained 10 PCF low-density cancellous, thin walled low-density cortical shell, overall length 45.5 cm, and 16 mm hollow canal.^18^‘Osteoporotic femur’ models are intended to mimic the specific biomechanical properties of a real osteoporotic femur and were selected since they were likely to more closely match those in the cadaveric testing group. Pre-operative implant size selection and neck cut to recreate preoperative offset and leg length was planned using proprietary software (IMPAX Orthopaedic Tools, Agfa Healthcare) following plain anteroposterior radiographs with a 25 mm diameter scaling ball. Preparation and implantation was performed according to manufacturer’s guidance by a single experienced surgeon (JL) to minimize variability.

After preparation, distal femoral resection was performed so that 40 mm of specimen remained between the stem tip and the distal fixative. Specimens were fixed into steel pots using a rapid setting resin fixative (cadaveric femurs: polymethylmethacrylate and composite femurs: G&B Epoxy Acrylate Resin, G&B Fissaggi, UK) in an identical alignment to those in the cadaveric group (six degrees of valgus in the coronal plane). Femurs were implanted with an appropriately sized fully coated cementless femoral stem with and without a medial calcar collar (Corail, DePuy Synthes, Leeds UK) in accordance with manufacturer guidelines and underwent visual inspection (composite femurs) or CT (cadaveric specimens) to screen for intraoperative fractures.

### Experimental setup

**Figure 1.**
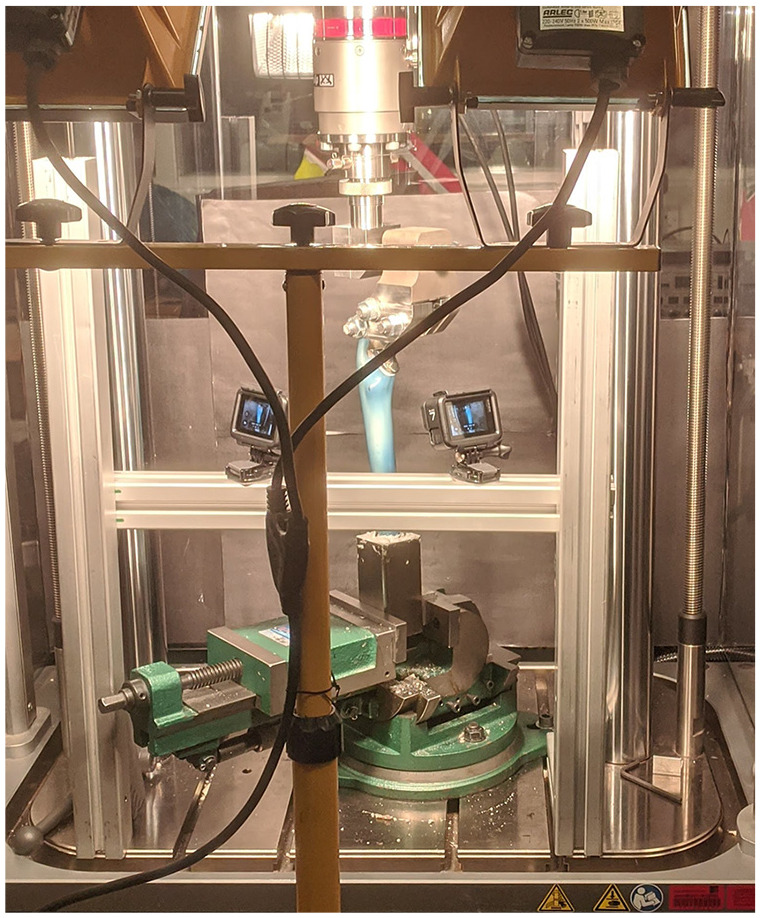
Experimental setup.

The test set up was adapted from previous methods^11^ and details of this adaptation have been previously published.^6^ In the adapted method the femoral stem had a 36 mm femoral head attached to the trunnion which was then held in a custom clamp (Figure 1). The specimen was subjected to a vertical load of 1500N for 10 s and then an additional rotational displacement of 45° in 1 s. An axialrotational method was chosen because this has previously been shown to reproduce PFF at the level of the stem^10,11,16^ and may be a common mechanism of injury in early PFF around cementless stems.

For composite femora tests, the potted specimen was secured distally into a clamp which was secured to the base of the materials testing machine and the specimen position was adjusted in two planes to ensure precise positioning. Simulated PFF were conducted using an identical loading regimen in a material testing machine (cadaveric femurs: MTS 858.2; Eden Prairie, MN, USA and composite femurs: ElectroPuls E10000, Instron, USA). In composite femur trials rotation was applied directly to the femoral head using a custom clamp that additionally ensured that the rotation axes was aligned to the femoral axes.

Fracture torque and rotational displacement were measured and torsional stiffness (rotary displacement divided by torque) and rotational work prior to fracture were estimated (area under rotatory displacement torque curve). Fractures were classified according to the unified classification system (UCS)^20^ and each trial was recorded to establish fracture mechanism using video camera equipment (cadaveric GoPro 4, GoPro, California, USA and composite femurs used GoPro Hero 8, GoPro, California, USA).

### Statistical methods

Data were tested for normality using a Shapiro-Wilks test and comparisons between cadaveric and composite femur groups were conducted using a Mann-Whitney U test, with significance set at *p* < 0.05. Comparisons were stratified by implant collar, since this has been shown to affect the mechanical properties prior to fracture.^5,6^

## Results

The baseline demographics for cadaveric femur donors are shown in Table 1. Results demonstrated statistically similar values for fracture torque, fracture displacement and torsional stiffness for cadaveric and composite femurs (Table 2). Results in the cadaveric tests displayed a greater variability in results versus composite femur results. There was a non-significant trend for a greater rotational displacement at fracture in the cadaveric groups but this failed to reach statistical significance. This observation lead to a non-significant decrease in torsional stiffness for all cadaveric specimens (*p* range 1.0 to 0.1) and a significantly greater rotational work prior to fracture in cadaveric versus composite femurs (collarless stems: 10.51 [9.71 to 12.57] vs 5.21 [4.25 to 6.04], *p* = 0.01 and for collared stems: 15.38 [14.01 to 17.05] vs 5.76 [4.92 to 6.64], *p* = 0.01).

**Table 1.** Basic demographics of cadaveric femur donors.

	Result
*n*	9
Age (median [IQR])	76.00 [69.00 to 81.00]
Height (median [IQR])	158.00 [157.00 to 167.00]
Female sex *n* (%)	9 (100%)
BMD (Range)	1.08 to 1.34 gHA/cm^3^

**Note**: IQR denotes interquartile range, BMD is bone mineral density and gHA/cm^3^ is grams of hydroxyapatite per cubic centimetre.

**Table 2.** Comparison of biomechanical results between trials conducted with cadaveric and composite femur specimens.

		Group		
Implant		Composite femur	Cadaveric femur	*p*
Collarless	*n*	6	4	
	Rotational displacement at fracture in Rad(median [IQR])	0.33 [0.32, 0.34]	0.44 [0.41, 0.46]	0.20
	Torque at fracture in Nm (median [IQR])	45.12 [39.13, 48.09]	41.91 [35.67, 51.35]	0.67
	Rotational work in Joules (median [IQR])	5.21 [4.25, 6.04]	10.51 [9.71, 12.57]	0.01*
	Torsional stiffness in Nm/rad (median [IQR])	138.79 [122.53, 140.59]	113.33 [74.46, 151.52]	1.00
Collared	*n*	6	5	
	Rotational displacement at fracture in radians(median [IQR])	0.29 [0.27, 0.31]	0.50 [0.37, 0.55]	0.07
	Torque at fracture in Nm (median [IQR])	48.41 [42.60, 50.27]	48.63 [44.62, 58.61]	0.72
	Rotational work in Joules (median [IQR])	5.76 [4.92, 6.64]	15.38 [14.01, 17.05]	0.01*
	Torsional stiffness in Nm/rad (median [IQR])	158.36 [152.61, 163.54]	147.05 [97.41, 153.03]	0.10

*Note*. IQR is interquartile range. * indicates statistical significance at *p *< 0.05.

**Figure 2.**
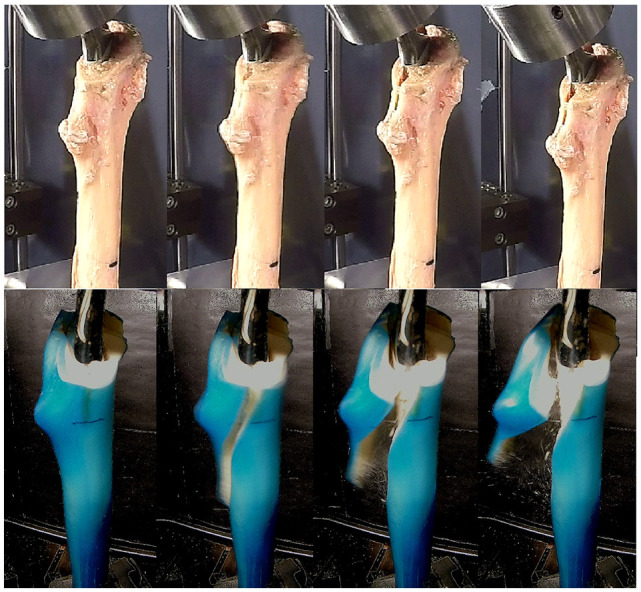
Comparison of collarless fracture pattern between human cadaveric specimens (top row) and osteoporotic sawbones (bottom row). Fracture occur in a similar position on the proximal femur. Fracture fragment acceleration is noticeably less in the cadaveric versus composite models.

**Figure 3.**
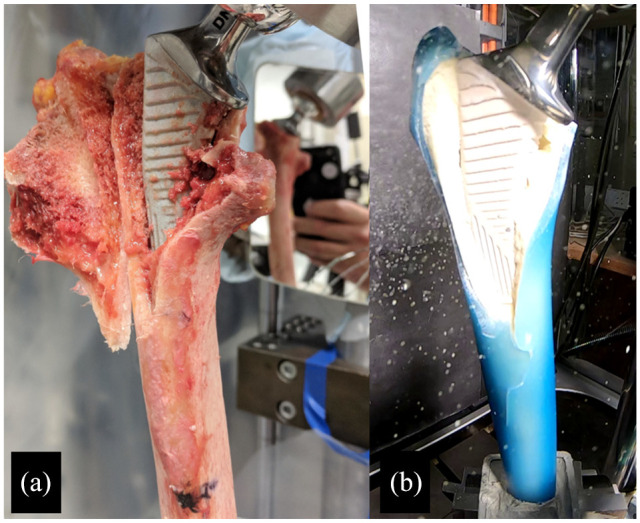
An example of fracture patterns which occurred after collared cementless stem trials with cadaveric specimen (A) and composite femur specimen (B).

Fracture resulted in UCS B2 fractures in all trials. Subjective assessment of fracture pattern demonstrated similar patterns of fracture in cadaveric and composite femur testing (Figures 2 and 3), although there was subjectively greater velocity of the fracture fragments from the composite femur specimens in comparison to the cadaveric specimens.

## Discussion

This study has demonstrated comparable fracture torque and fracture patterns between composite femur and cadaveric femur trials. Rotational work in cadaveric femurs was greater than that recorded in composite femurs with the same loading regimen. This was largely because cadaveric femur trials fractured at a greater median rotational displacement but this difference did not reach statistical significance.

This study confirms that the results obtained with composite femur specimens are largely comparable to those results from testing using cadaveric femur trials in this study and elsewhere using similar methods,^5^ with some important differences. Whilst the fracture torque was comparable between composite and human femurs the composite femurs appear to be stiffer than cadaveric counterparts and fracture occurred at smaller angular displacements in comparison to cadaveric specimens. Whole human femur stiffness follows a rate dependent relationship,^21^ with strength and stiffness increasing with loading rate. The stiffness of whole composite femurs has been found to be constant over a range of loading rates^22^ and comparable to human specimens in torsional and axial loading.^17^ When the stem is placed under axial load, the stem can move independently of the femur under high loading rates^5,6^ and the implant-femur construct stiffness is dependent on the mechanical properties of the stem, the stem-bone interface and the bone. The internal foam of the composite femur is homogenous and does not represent the variation seen in mechanical strength and mineral density within human femurs.^23^ This may make the stiffness of foam adjacent to the stem greater than that which is seen in human specimens and reduce relative stem-femur displacement under high load conditions. In addition the coefficient of friction between a stem and artificial bone is dissimilar to human trabecular bone^24^ and may lead to differing results when loads are transferred across the stem-bone interface. During preparation of the cadaveric femurs it was noted that the foam did not behave in a similar way to normal trabecular bone. The Corail hip system uses an impaction broaching technique to prepare the femur for implantation.^25^ The broaching technique was not easy to replicate in the composite femur and the foam did not appear to compress against the broach in a similar way. In addition, foam particles which are broached tended to fall into the void in the central portion of the composite femur specimen. Absence of a compressed trabecular layer in the composite femur is likely to change the stem-bone interface mechanics and may account for some differences in rotational stiffness seen in this study.

Previous studies assessing neck of femur fracture patterns in composite femur models have found fracture patterns which are both consistent with cadaveric and embalmed femurs^13,26^ unrealistic patterns^27^ and also unrealistic stability when the mechanical properties of ‘fixed’ composite femur fractures are tested.^28^ In this study, the pattern of fracture between composite and human femurs in an axial loading model was very similar and in agreement with a small study including just one cadaveric trial.^13^ This would suggest that the failure mechanism is similar between composite and human femurs during axial loaded PFF simulations.

For researchers hoping to use similar composite femur specimens mentioned in this study it is worth commenting on the practical constraints experienced by the authors. The Osteoporotic composite femur specimens had a very thin cortical shell which in comparison to the cancellous foam were incredibly fragile and in future work, care should be taken during manipulation, preparation and implantation of the femur since inadvertent fracture is much more likely than standard composite femurs which represent adult anatomy.

The main limitation in this study is small sample sizes in both cadaveric and composite femur groups. Given the precious resource which cadaveric samples represent, this is a common drawback of biomechanical testing. Reassuringly the results of the composite femur tests are also similar to other results, which used a similar, although non-identical methodology.^5^ The small sample sizes reduce the power of our comparisons and further work should seek to validate composite femur models with larger sample sizes. This study utilised two different fixatives, which may have contributed to the variation between experiments. Although the loading methodology has been used widely in previous work, further work should seek to improve loading conditions to maximise repeatability and validity to typical periprosthetic fractures. We were not able to quantify relative motion between stem and femur, which would have enabled interesting comparison of implant behaviour during rotational loading. Future work should seek to integrate methods which allow accurate quantification of implant displacement and foam deformation. This study only compares the results with a torsional loading model. Even though this model has been used previously and produces clinically valid fracture patterns around cementless stems, testing should also look to validate the use of composite femurs with a range of loading methods.

Given the reduced variability of results and comparable fracture torque and the similarity in fracture patterns from the fracture trials using composite samples versus cadaveric femurs, the use of composite femur models is a reasonable choice where maximum fracture torque is the outcome of choice.
